# Inter-neuronal signaling mediated by small extracellular vesicles: wireless communication?

**DOI:** 10.3389/fnmol.2023.1187300

**Published:** 2023-04-27

**Authors:** Damaris Nieves Torres, Sang H Lee

**Affiliations:** ^1^Department of Pharmacology and Toxicology, Medical College of Wisconsin, Milwaukee, WI, United States; ^2^Neuroscience Research Institute, Medical College of Wisconsin, Milwaukee, WI, United States

**Keywords:** extracellular vesicles, exosomes, neuron–neuron communication, synapse-independent, neuronal plasticity

## Abstract

Conventional inter-neuronal communication conceptualizes the wired method of chemical synapses that physically connect pre-and post-synaptic neurons. In contrast, recent studies indicate that neurons also utilize synapse-independent, hence “wireless” broadcasting-type communications *via* small extracellular vesicles (EVs). Small EVs including exosomes are secreted vesicles released by cells and contain a variety of signaling molecules including mRNAs, miRNAs, lipids, and proteins. Small EVs are subsequently absorbed by local recipient cells *via* either membrane fusion or endocytic processes. Therefore, small EVs enable cells to exchange a “packet” of active biomolecules for communication purposes. It is now well established that central neurons also secrete and uptake small EVs, especially exosomes, a type of small EVs that are derived from the intraluminal vesicles of multivesicular bodies. Specific molecules carried by neuronal small EVs are shown to affect a variety of neuronal functions including axon guidance, synapse formation, synapse elimination, neuronal firing, and potentiation. Therefore, this type of volume transmission mediated by small EVs is thought to play important roles not only in activity-dependent changes in neuronal function but also in the maintenance and homeostatic control of local circuitry. In this review, we summarize recent discoveries, catalog neuronal small EV-specific biomolecules, and discuss the potential scope of small EV-mediated inter-neuronal signaling.

## 1. Introduction

EVs are traditionally classified based on their size, cargo molecules, and originating cell populations (Fowler, 2019). EVs are called by many names − exosomes, microvesicles, ectosomes, shedding vesicles, microparticles, etc. Exosomes (50–200 nm in diameter) are derived from the intraluminal vesicles (ILVs) of multivesicular bodies (MVBs). Ectosomes, also called microvesicles, are generated by the shedding of the direct outward budding of the plasma membrane, whose sizes are in the range of ~100 nm to 1 μm in diameter. However, as EVs are highly heterogenous, even at the single cell levels (Zabeo et al., 2017), it has become harder to define specific categories. In this minireview, we use a term small EVs for EVs smaller than 200 nm in diameter (Thery et al., 2018), which include exosomes.

Exosomes were first discovered nearly 40 years ago and had long been considered as a mere cellular waste disposal mechanism (Trams et al., 1981; Thery, 2011). However, recent major discoveries argue their role as active messengers for cellular communication: exosomes contain many bioactive molecules (especially mRNA and microRNAs) that are re-uptaken by cells (Valadi et al., 2007). Since then, the signaling function of EVs are well established in immune responses and cancer cell biology (Thery et al., 2009).

The vertebrate brain contains various types of non-neuronal cells including astrocytes, oligodendrocytes, and microglia. It is well known that small EVs and exosomes secreted from these non-neuronal cells affect various neuronal functions. Since the first demonstration that postmitotic neurons secrete exosomes by Sadoul’s group (Faure et al., 2006), significant advances have been made in neuronal EV research (Budnik et al., 2016; Fowler, 2019). Now, neuronal exosomes are implicated in a variety of processes including neurogenesis, axon guidance, synaptogenesis, synapse elimination, neuroprotection, mRNA expression, synaptic plasticity, and inflammation (Korkut et al., 2009; Escudero et al., 2014; Gong et al., 2016; Lee et al., 2018; Sharma et al., 2019; Vilcaes et al., 2021; Antoniou et al., 2023). Brain EVs are also implicated for the spread of pathogenic molecules such as amyloid β (Aβ), amyloid precursor proteins (APP), prions, tau, and α-synuclein, which a recent review extensively covered (Delpech et al., 2019). Also, we note that EVs are actively developed as biomarkers for specific diseases and vehicles for drug delivery. However, in this review, we will limit our discussion to the recent discoveries on the signaling function of neuronal small EVs under normal healthy conditions, focusing specifically on cargo molecules of small EVs.

## 2. Signaling molecules present in neuronal EVs and their functions

**Figure 1:**
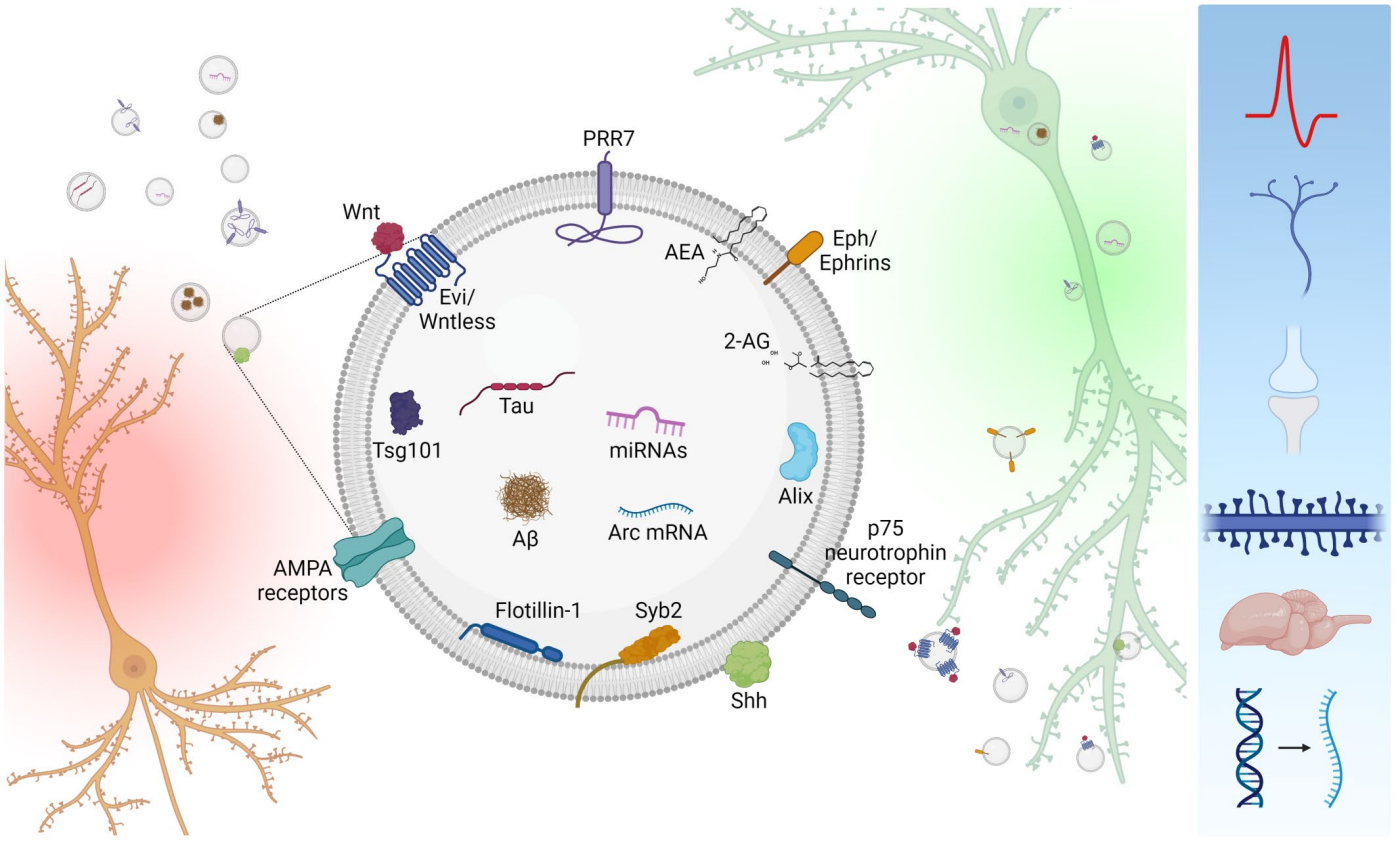
Inter-neuronal signaling mediated by neuronal small EVs. Neurons (*orange*) secrete small EVs carrying a variety of signaling molecules (*center*). However, neuronal small EVs are heterogenous in size and molecular composition, and single small EVs unlikely carry all these molecules. Secreted small EVs are absorbed by recipient neurons (*green*) either *via* endocytosis or membrane fusion. Uptaken small EVs affect a variety of cellular processes including neuronal firing, axon guidance, synapse formation, dendritic spine maintenance, synapse elimination, brain development, and mRNA expression (*right blue box*). Created with BioRender.com.

Since the discovery that Wnts are secreted on EVs (Korkut et al., 2009), more than a dozen of signaling proteins and other molecules (miRNAs, mRNAs, and lipid messengers) have been identified to be secreted *via* EVs by neurons (see Table 1; Figure 1) and the number is only expected to increase (Chen et al., 2022).

**Table 1:** Signaling and pathogenic molecules carried on neuronal exosomes.

Molecules	Organism/neuron	Function/Exosome-specific function	Reference	Note
**Proteins**				
Alpha-synuclein (α-syn)	Mouse, cortical neurons	Synaptic vesicle trafficking, PD pathology	Danzer et al. (2012)	Exosomes containing α-synuclein contribute to the neuron-to-neuron spread of toxic α-syn.
Amyloid β	Human, neocortical and IPSC neurons; Mouse, cortical neurons	AD pathology	Yuyama et al. (2012), Sardar Sinha et al. (2018)	Exosomes carrying Aβ contribute to spread of AD pathology and promote microglial clearance.
AMPA receptors	Rat, cortical neurons; Rat, hippocampal neurons	Synaptic transmission/ exosomal function unknown	Faure et al. (2006), Lachenal et al. (2011)	Cultured neurons release exosomes containing GluR2/3 in an activity-dependent manner.
APP, APP C-terminal fragment	Mouse, cortical neurons; Rat, cortical neurons	AD pathology	Laulagnier et al. (2018), Miranda et al. (2018)	Exosomes released by neurons act as carriers and propagators of APP and its C-terminal fragment.
Eph/Ephrins	Mouse, cortical neurons	Axon guidance	Gong et al. (2016)	Eph/Ephrin-containing exosomes (EVs) disrupt repulsive axon guidance and cause growth cone collapse.
Sonic hedgehog (Shh)	Mouse, Purkinje cells, Cortical pyramidal neurons	Embryonic brain development	Coulter et al. (2018)	Shh is secreted on EVs *via* Chmp1a-dependent manner.
Presenilin	Human, IPSC neurons	Subunit of protease complex	Podvin et al. (2021)	Mutant presenilin within exosomes modifies the proteomic cargo of these exosomes.
Prion protein	Mouse, hypothalamic neuron cell line; Mouse, hypothalamic neurons	Misfolding of proteins, neurodegene-rative diseases	Vella et al. (2007), Guo et al. (2015)	Packaging of prion protein into exosomes relies on N-terminal modification and the neutral sphingomyelinase pathway.
PRR7	Rat, hippocampal neurons	Wnt inhibitor, synapse elimination	Lee et al. (2018)	PRR7 secreted on exosomes blocks Wnt signaling and promotes excitatory synapse loss.
p75 neurotrophin receptors	Rat, sympathetic neurons	Neurite outgrowth, neuronal survival	Escudero et al. (2014)	p75 is trafficked through MLVs and released on exosomes upon depolarization.
Synaptobrevin 2 (VAMP2)	Rat, hippocampal neurons; Mouse, hippocampal neurons	Augment inhibitory synaptic transmission	Vilcaes et al. (2021)	Synaptobrevin present in small extracellular vesicles promotes inhibitory neurotransmission.
Tau	Rat, cortical neurons; Human, IPSC neurons	Microtubule stabilization, AD pathology	Reilly et al. (2017), Wang et al. (2017), Guix et al. (2018), Winston et al. (2018)	Tau is secreted on exosomes and potentially contributes to the spread of AD pathology.
Wnts/Evi/ Wntless	*Drosophila*, motor neurons; Rat, hippocampal neurons	Synaptogenesis and synapse maintenance	Korkut et al. (2009), Lee et al. (2018)	Wnt proteins secreted on exosomes are involved in NMJ development (in Drosophilia) and synapse maintenance.
**RNA species**				
Arc mRNA	*Drosophila*, motor neurons; Rat, hippocampal neurons	Synaptic plasticity	Ashley et al. (2018), Pastuzyn et al. (2018)	Arc-encapsulated mRNA is transferred to target cells by exosomes.
miR-124	Mouse, primary cortical neurons	Microglial activation	Veremeyko et al. (2019)	Exosomes containing miR-124 modulate microglial activity.
miR-21-5p	Mouse, dorsal root ganglion neurons; Mouse, hippocampal cell line; Rat, cortical neurons	Inflammation	Simeoli et al. (2017)	DRG neurons release exosomes containing miR-21-5p induce pro-inflammatory responses in macrophage.
miR-132	Rat, cortical neurons	Post-transcriptional regulation of gene expression	Xu et al. (2017)	Neuron-derived exosomes containing miR-132 maintain brain vascular integrity.
miR-124-3p	Mouse, primary cortical neurons	Post-transcriptional regulation of gene expression	Men et al. (2019)	Neuron-derived exosomes containing miR-124-3p alters GLT1 gene expression in astrocytes.
miR-132-5p, miR-218-5p, and miR-690	Mouse, primary cortical neurons	Synaptic plasticity	Antoniou et al. (2023)	BDNF induces the secretion of neuronal small EVs containing miRNAs to affects synapse formation and synaptic activity in recipient neurons.
**Lipid messengers**				
2-AG, AEA	Mouse, midbrain DA neurons	Disinhibition of DA neurons	Nakamura et al. (2019)	Cocaine triggers the release of small EVs containing 2-AG, promoting neuronal disinhibition.

### 2.1. Wnts and Evi/Wntless

Wnt signaling controls myriads of fundamental biological processes during development and adult life (Clevers and Nusse, 2012). Wnts are one of the first molecules identified to be secreted on EVs and has signaling function during development (Greco et al., 2001; Korkut et al., 2009; Gross and Boutros, 2013). Wnts are hydrophobic protein and thus its secretion mechanism has been in question. EVs serve as carriers for the extracellular dissemination of Wnts (Zhang and Wrana, 2014). In *Drosophlia* neuromuscular junction (NMJ), EVs containing Wnts and Wnt carrier protein Evi/Wntless are involved in the development of NMJs (Korkut et al., 2009). Wnt signaling is also important for synaptogenesis, synapse and dendritic maintenance as well as spatial learning and memory (Dickins and Salinas, 2013; Chen et al., 2017). Recent studies indicate a critical role for Wnts in the formation of hippocampal long-term potentiation (LTP) (McLeod et al., 2018), raising an important question as to whether the effect is mediated by exosomal Wnts.

### 2.2. Proline rich 7

Proline rich 7 (PRR7) is a proline-rich type 1 transmembrane protein, first identified as a protein enriched in the postsynaptic density (Murata et al., 2005). Recent studies indicate that PRR7 is secreted by neurons on exosomes in an activity-dependent manner and functions as a novel Wnt inhibitor in synapse regulation by inhibiting Wnt secretion on exosomes (Lee et al., 2018). Importantly, `exosomes carrying high levels of PRR7 are absorbed by neurons and were shown to be necessary and sufficient to induce excitatory synapse loss in recipient neurons. These findings highlight the signaling function of neuronal exosomes in synapse maintenance in the central neurons. The zebrafish ortholog of *PRR7*, *Ottogi* (*Otg*), also functions as a Wnt inhibitor during development (Kim et al., 2017). Otg inhibits Wnt signaling by blocking the surface expression of Frizzled receptors. Interestingly, Otg only has cell-autonomous effects, suggesting that it may not be secreted on exosomes.

### 2.3. p75 neurotrophin receptor

The neurotrophin receptor p75, also known as nerve growth factor receptor, mediates multifaceted signaling pathways of neurite outgrowth, neuronal survival, and death (Ibanez and Simi, 2012). One study showed that p75 undergoes endosomal trafficking to MVBs and is subsequently released by exosomes (Escudero et al., 2014). These authors suggested an interesting possibility of transferring p75 signaling complexes from one cell to another. However, it remains to be determined whether exosomal p75 can induce neurotrophin signaling in recipient cells.

### 2.4. Eph and ephrins

Eph receptor tyrosine kinase and their membrane-tethered ephrin ligands play crucial roles in axon guidance and specific synapse formation (Klein and Kania, 2014). Interestingly, Gong et al., showed that dissociated motor cortex neurons secrete biologically active Eph and ephrins on exosomes that can cause growth cone collapse, suggesting the potential role of exosomal Eph and ephrins in neuronal development and synapse physiology (Gong et al., 2016).

### 2.5. AMPA receptors

AMPA receptors are major glutamate-gated ion channels and the main excitatory postsynaptic potential generator in the brain (Bassani et al., 2013). Thus, AMPA receptors control synaptic transmission and are the main substrates of synaptic plasticity (Shepherd and Huganir, 2007; Kessels and Malinow, 2009). Interestingly, unlike NMDA receptors, AMPA receptors are one of the consistently found molecules in neuronal exosome preparations (Faure et al., 2006; Lachenal et al., 2011; Lee et al., 2018). These findings suggest that exosomal AMPA receptors may contribute to neuronal excitability of recipient neurons. However, currently the role of exosomal AMPA receptors are completely unknown.

### 2.6. Synaptobrevin 2

Synaptobrevin 2 (Syb2; also called VAMP2) belongs to N-ethylmaleimide-sensitive factor (NSF)-attachment factor receptor (SNARE) proteins that are involved in synaptic vesicle fusion. Syb2 was found from small EV preparations purified from culture supernatant of cultured rat hippocampal neurons, along with other SNARE proteins, syntaxin-1 and synaptotagmin-5 (Vilcaes et al., 2021). Remarkably, the authors showed that small EVs containing syb2 are absorbed by neurons and selectively enhance inhibitory synaptic transmission in a CD81-dependent manner, indicating that neuronal EVs can potentially regulate the excitability of neurons. However, it remains to be determined whether the exosomal secretion of syb2 is a regulated (activity-dependent) process.

### 2.7. Arc protein and mRNA

The activity-regulated cytoskeleton associated protein (Arc) is a neuron-specific protein that is critical for synaptic plasticity and memory formation (Korb and Finkbeiner, 2011). Two groups found that Arc mRNA is encapsulated in retrovirus-like gag protein Arc and secreted *via* small EVs for trans-synaptic traffic in synaptic boutons of the neuromuscular junction or inter-neuronal RNA transfer (Ashley et al., 2018; Pastuzyn et al., 2018). Although small EV-carried Arc mRNAs are shown to be functionally active, their contribution to synaptic plasticity awaits further investigation.

### 2.8. microRNAs

MicroRNAs (miRNAs) are short (19–24 nucleotide) noncoding single-stranded RNAs that function in post-transcriptional gene silencing (Saliminejad et al., 2019). Goldie et al. first showed that depolarized SH-SY5Y human neuroblast cells secrete miRNAs in exosomes (Goldie et al., 2014). Since then, several reports indicate that neurons also secrete various species of miRNAs including miR-124, miR-21-5p, and miR132, which are subsequently absorbed by microglia, astrocytes, or endothelial cells. These exosome-derived miRNAs influence a variety of processes in the recipient cells, including the modulation of microglial activity (Veremeyko et al., 2019), pro-inflammatory responses (Simeoli et al., 2017), gene transcription in astrocytes (Men et al., 2019), and brain vascular integrity (Xu et al., 2017). Most recently, it was shown that brain-derived neurotrophic factor (BDNF) promotes the sorting of miRNAs to neuronal exosomes, which enhances excitatory synapse formation in recipient neurons (Antoniou et al., 2023).

### 2.9. Endocannabinoids

Endocannabinoids (eCBs) are lipid messengers that modulate synaptic functions in both short-term and long-term forms of plasticity (Castillo et al., 2012). Interestingly, it was reported that microglia secrete small EVs containing N-arachidonylethanolamine (AEA) on their surface (Gabrielli et al., 2015). The AEA-containing small EVs can induce type 1 eCB receptor (CB1)-mediated signaling and inhibit presynaptic transmission, suggesting an active signaling function of small EV-carried AEA. Moreover, more recent studies showed that cocaine induces the secretion of another form of eCB, anandamide (2-AG), *via* small EVs in the midbrain of mice (Nakamura et al., 2019). It is unclear at this point whether neurons also secrete AEA and 2-AG on EVs. However, since neurons actively synthesize eCBs and eCBs have such profound neuromodulatory function on neurons, further studies on the possibility are warranted.

## 3. Neuronal EV secretion mechanisms

Importantly, exosome secretion by neurons could be a regulated process that is induced by high K^+^-induced depolarization, GABA_A_R antagonists, and/or blocked by NMDA receptor blockers (Faure et al., 2006; Lachenal et al., 2011; Wang et al., 2017; Lee et al., 2018; Kumar et al., 2020), indicating that it is an activity-dependent phenomenon and thus has signaling function. We also note here that neuronal EV secretion could also be a constitutive process not dependent on activity (Vilcaes et al., 2021).

Studies done in other types of cells indicate that multiple routes for ILV biosynthesis exist (Blanchette and Rodal, 2020), including well-studied the Endosomal Sorting Complex Required for Transport (ESCRT)-dependent process and ceramide/lipid-dependent process (Trajkovic et al., 2008; Babst, 2011). In the case of neuronal exosomes and small EVs, cargo molecules such as Eph, ephrins and Shh are shown to be dependent on ESCRTs for their EV secretion. Interestingly, ESCRT-III protein Chmp1a is specifically required for the secretion of Shh *via* small EVs (Coulter et al., 2018). On the other hand, small EV secretion of neuronal miRNAs was shown to be dependent on ceramide (Antoniou et al., 2023). These findings suggest that different cargo molecules undergo distinct exosomal sorting pathways that require specific ESCRT proteins and/or lipids in neurons.

Rab GTPases play critical function in vesicular traffic inside cells (Stenmark, 2009). It was shown that different species of Rab proteins are required for the exosomal secretion of specific cargo proteins in neurons: for example, Wnts require Rab11 but not ceramide (Koles et al., 2012; Beckett et al., 2013). On the other hand, the exosomal secretion of PRR7 requires Rab27b not Rab11 (Lee et al., 2018).

Neuronal exosomes are shown to be released at soma and dendrites (Lachenal et al., 2011; Kumar et al., 2020), which is consistent with the MVB localization in neurons (Von Bartheld and Altick, 2011). However, it is worth to point out that there remains a possibility of axonal release since small EVs carrying Wnts are secreted at axon terminals in the neuromuscular junctions of *Drosophilia* (Korkut et al., 2009).

Membrane fusion events are controlled by Ca^2+^. Likewise, neuronal exosome secretion was also shown to require Ca^2+^ (Lachenal et al., 2011). However, it remains to be identified what specific steps of exosome secretion are controlled by Ca^2+^: for example, initial endocytic events of cargo molecules and/or exocytosis processes required for the membrane fusion of MVBs to the PM. Interestingly, the knockdown of VAMP3 specifically attenuated fibroblast growth factor 2-induced neuronal exosome release (Kumar et al., 2020), suggesting that specific SNARE proteins might be involved in the EV secretion.

## 4. Neuronal EV uptake mechanisms

In contrast to exosome secretion mechanisms, the mechanisms by which neurons uptake neuronal exosomes are much less understood. Several studies suggest that EV uptake depends on both the origin of EVs and recipient cells (Fowler, 2019). Interestingly, neuronal exosomes seem to be preferentially uptaken by neurons, indicating their aptitude for inter-neuronal communication. For example, studies using GFP-fused tetanus toxin C-terminal protein showed that neuronal exosomes are uptaken and endocytosed specifically by neurons and not by astroglia cells (Chivet et al., 2014). Moreover, our studies using PRR7-containing neuronal exosomes also showed preferential absorption by neurons through a process of membrane fusion (Lee et al., 2018). Similarly, APP-and Syb2-conbtaining neuronal exosomes were also shown to undergo membrane fusion (Laulagnier et al., 2018; Vilcaes et al., 2021). On the other hand, EVs containing miRNAs are uptaken by neurons in a dynamin-dependent manner, suggesting endocytosis-dependent mechanism (Antoniou et al., 2023). Therefore, it seems neurons uptake exosomes by both endocytosis and membrane fusion mechanisms. However, it remains to be identified whether specific cargo-molecules in exosomes and neuronal surface molecules mediate the selective neuronal uptake of neuronal exosomes *via* different mechanisms.

Synaptic clefts of hippocampal neurons are ~24 nm in average width (Zuber et al., 2005), which is crowded with cell adhesion molecules and other receptors. Therefore, synaptic clefts seem too small for EV to freely enter and diffuse in. Considering this space limitation, neuronal exosomes are most likely uptaken at non-synaptic sites, including extra-synaptic membrane area in dendrites, axons, and soma. Further studies are necessary to determine the initial EV contact sites of neurons, also where the endocytosis and/or membrane fusion events are initiated.

## 5. Extracellular space (ECS) and small EV signaling range

Neurons and other cells in the brain are separated by ECS. ECS occupies almost one-fifth of brain volume and serves as a reservoir of ions and a physical corridor for diffusional transport of substances including nutrients (Hrabetova et al., 2018). Since EVs are secreted to ECS, for them to move freely around and spread in the brain tissue, intercellular space should be larger than the size of EVs. Intriguingly, contrary to the conventional idea based on the images of fixed brain tissue observed under electron microscope, state-of-the-art imaging techniques reveal that ECS is larger than people previously thought (Korogod et al., 2015; Tonnesen et al., 2018). ECS size ranges between 80 and 270 nm (ave. 150 ± 40 nm) (Godin et al., 2017), which is big enough to allow exosomes or small EVs to move around in the brain. Moreover, ECS is not static but rather dynamic, and changes during neuronal activity, sleep, and disease courses (Binder et al., 2004; Ding et al., 2016; Hrabetova et al., 2018).

The effective signaling ranges of extracellular signaling molecules can be juxtacrine (direct neighbors), paracrine (over several cells), or long-range endocrine (Muller and Schier, 2011). Considering all findings on the brain ECS and neuronal EVs, it is likely that neuronal small EVs under normal conditions are absorbed by the immediate neighboring neurons (juxtacrine) and in an autocrine manner. Under certain conditions that allow the expansion of ECS and/or excessive secretion, small neuronal EVs diffuse further away before they are uptaken by neurons. In support of this long-range signaling, exosomes injected either directly into the brain or peripherally were found throughout the brains (Alvarez-Erviti et al., 2011; Yang et al., 2021). On the other hand, aging causes the shrinkage of ECS and thus may limit small EV travel, hampering the exosome-mediated intercellular communication.

## 6. Future directions

In summary, EVs, especially small EVs including exosomes, provide novel mechanisms for synaptic connection-independent inter-neuronal communication, which affect diverse neuronal functions. However, many important questions remain.

1. It is known that not all exosomes are created equal and rather diverse subpopulations of exosomes are released by the same cells (Bobrie et al., 2012; Zabeo et al., 2017), indicating heterogeneity of EVs. Then, do single neurons also release discrete populations of EVs that contain different cargo molecules for signaling purposes? Then, how diverse are the molecular compositions of single EVs?
2. Do individual neurons utilize multiple distinct mechanisms for the secretion of exosomes containing different cargos?
3. What are the mechanisms by which neuronal exosomes are specifically absorbed by neurons? Is this uptake process also activity-dependent or constitutive?
4. Lastly, what are the physiological functions of molecules carried on EVs?
  Almost all, if not all, molecules found in EVs are abundantly present inside cells and not specific to EVs. Therefore, it is very difficult to understand the specific function of these EV-carried molecules, distinguished from their cell-autonomous roles. Considering that most of EV studies used a large amount of purified but heterogenous EVs for the examination of their biological effects, future studies should be focused on the molecule-specific role in EV-mediated communication.

Answering these questions likely requires some technical innovations that allow singe EV proteomics and RNAseq studies. Despite these challenges, better understating of small EV-mediated inter-neuronal communication will reveal another important layer of plasticity mechanisms operating in the brain.

## Author contributions

DN and SL wrote and edited the manuscript and prepared the Figure illustration. All authors contributed to the article and approved the submitted version.

## Funding

The works in the Author’s lab are supported by NIH MH119105 and AG073610 grant (to SL).

## Conflict of interest

The authors declare that the research was conducted in the absence of any commercial or financial relationships that could be construed as a potential conflict of interest.

## Publisher’s note

All claims expressed in this article are solely those of the authors and do not necessarily represent those of their affiliated organizations, or those of the publisher, the editors and the reviewers. Any product that may be evaluated in this article, or claim that may be made by its manufacturer, is not guaranteed or endorsed by the publisher.
